# Immunoinformatic exploration of a multi-epitope-based peptide vaccine candidate targeting emerging variants of SARS-CoV-2

**DOI:** 10.3389/fmicb.2023.1251716

**Published:** 2023-10-17

**Authors:** K. M. Kumar, Yalpi Karthik, D. Ramakrishna, S. Balaji, Sinosh Skariyachan, T. P. Krishna Murthy, Kunnathur Murugesan Sakthivel, Badriyah S. Alotaibi, Mustafa Shukry, Samy M. Sayed, Muntazir Mushtaq

**Affiliations:** ^1^Department of Bioinformatics, Pondicherry University, Pondicherry, India; ^2^Department of Studies and Research in Microbiology, Mangalore University, Chikka Aluvara, Kodagu, Karnataka, India; ^3^Biotechnology Department, Dayananda Sagar College of Engineering, Dr. C.D Sagar Centre for Life Sciences, Dayananda Sagar Institutions, Bengaluru, India; ^4^Centre for Incubation, Innovation, Research and Consultancy (CIIRC^®^), Jyothy Institute of Technology, Bengaluru, Karnataka, India; ^5^Department of Microbiology, St. Pius X College, Rajapuram, Kerala, India; ^6^Department of Biotechnology, Ramaiah Institute of Technology, Bengaluru, Karnataka, India; ^7^Department of Biochemistry, PSG College of Arts and Science, Coimbatore, Tamil Nadu, India; ^8^Department of Pharmaceutical Sciences, College of Pharmacy, Princess Nourah bint Abdulrahman University, Riyadh, Saudi Arabia; ^9^Physiology Department, Faculty of Veterinary Medicine, Kafrelsheikh University, Kafrelsheikh, Egypt; ^10^Department of Economic Entomology and Pesticides, Faculty of Agriculture, Cairo University, Giza, Egypt; ^11^Department of Science and Technology, University College-Ranyah, Taif University, Taif, Saudi Arabia; ^12^MS Swaminathan School of Agriculture, Shoolini University of Biotechnology and Management Sciences, Solan, Himachal Pradesh, India

**Keywords:** SARS-CoV-2, vaccine design, *in-silico* modeling, peptide-protein docking, immune response profiling, MD simulation

## Abstract

Many countries around the world are facing severe challenges due to the recently emerging variants of SARS-CoV-2. Over the last few months, scientists have been developing treatments, drugs, and vaccines to subdue the virus and prevent its transmission. In this context, a peptide-based vaccine construct containing pathogenic proteins of the virus known to elicit an immune response was constructed. An analysis of the spike protein-based epitopes allowed us to design an “epitope-based subunit vaccine” against coronavirus using the approaches of “reverse vaccinology” and “immunoinformatics.” Computational experimentation and a systematic, comprehensive protocol were followed with an aim to develop and design a multi-epitope-based peptide (MEBP) vaccine candidate. Our study attempted to predict an MEBP vaccine by introducing mutations of SARS-CoV-2 (Delta, Lambda, Iota, Omicron, and Kappa) in Spike glycoprotein and predicting dual-purpose epitopes (B-cell and T-cell). This was followed by screening the selected epitopes based on antigenicity, allergenicity, and population coverage and constructing them into a vaccine by using linkers and adjuvants. The vaccine construct was analyzed for its physicochemical properties and secondary structure prediction, and a 3D structure was built, refined, and validated. Furthermore, the peptide-protein interaction of the vaccine construct with Toll-like receptor (TLR) molecules was performed. Immune profiling was performed to check the immune response. Codon optimization of the vaccine construct was performed to obtain the GC content before cloning it into the *E. coli* genome, facilitating its progression it into a vector. Finally, an *in-silico* simulation of the vaccine–protein complex was performed to comprehend its stability and conformational behavior.

## Graphical Abstract

Schematic representation of study design. 
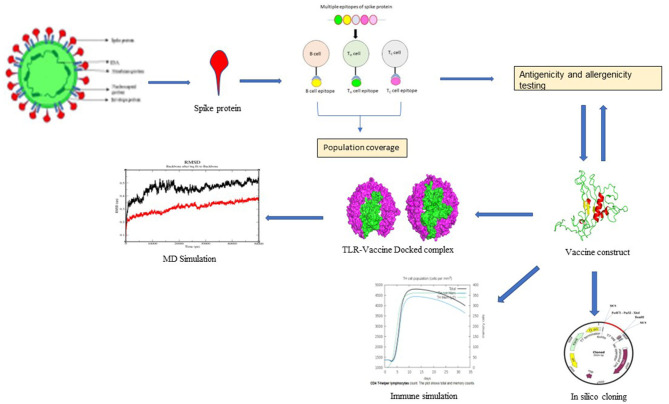

## 1. Introduction

COVID-19 (coronavirus disease-19), caused by the severe acute respiratory syndrome coronavirus 2 (SARS-CoV-2), was identified in Wuhan City, China, in late 2019 when unexplained cases of pneumonia were reported. The new strain of this virus was not spotted earlier in humans. In March 2020, the World Health Organization (WHO) proclaimed the novel coronavirus outbreak as a global pandemic (Abd El-Aziz and Stockand, 2020). The virus infected more than 31.6 million people across the globe, leading to more than 971,000 (3.07%) mortalities in 220 countries and territories after 6 months of its outbreak (Weber et al., 2020). Despite strict global constraints and quarantine efforts, the incidence of COVID-19 continues to rise. As of 27 May 2022, 525,467,084 confirmed casesCOVID-19 and 6,285,171 deaths have been reported by the WHO. The CoVs are classified into Alphacoronavirus (αCoVs), Betacoronavirus (βCoVs), Deltacoronaavirus (δCoVs), and Gammacoronavirus (γCoVs). The structural proteins of the coronavirus are spike protein (S), membrane protein (M), nucleocapsid protein (N), and envelope protein (E) (Satarker and Nampoothiri, 2020). The S-protein exhibits different degrees of conservation across the Coronaviridae family. It is a type-I transmembrane N-linked glycosylated protein (Yadav et al., 2021). It binds to the cellular membrane ACE2 receptor (Pohl et al., 2021) that permits the entry of the virus into the host cell and triggers a viral–host membrane fusion (Jaimes et al., 2020). The host cell proteases cleave the S-protein. It does so either following the attachment of virions to the host cell membrane or during virion maturation and exit, which is crucial for the S-protein's fusion function (Ou et al., 2020). The receptor-binding region (RBD) identifies the host ACE2 receptor. Thus, RBD is a crucial factor of virus–receptor interaction that considers host selectivity, virus tropism, and infectivity (Su et al., 2020). In late 2020, the emergence of variants posed an increased risk of transmission to global public health, leading to the classification of variants into variants of interest (VOIs) and variants of concern (VOCs) aligning them for global monitoring and research. Variants of concern include Alpha, Beta, Delta, Gamma, and Omicron. Variants of interest include Kappa, Lambda, Iota, and Epsilon. Several antiviral drugs and treatment agents such as favipiravir, hydroxychloroquine, corticosteroids, chloroquine, monoclonal antibodies, and convalescent plasma have been assessed for treating COVID-19. However, they have not been found to be effective (Gavriatopoulou et al., 2021).

Contrarily, vaccines have so far proven to be the best agents in preventing COVID-19. Vaccines consist of macromolecules from microorganisms or attenuated and killed whole microorganisms that are used to induce an immune response. Moreover, DNA vaccines contain DNA that codes for a protein to induce an immune response. At present, the availability of genomic and proteomic data, advanced software, and immunological datasets considerably assists researchers in detecting the potent epitopes from antigens that can be used to develop active peptide-based vaccines (De Gregorio and Rappuoli, 2012; Kalita et al., 2019). Fragments of antigenic proteins of pathogens are used in peptide-based vaccines to elicit natural defenses against the target pathogen (Saadi et al., 2017). In recent studies, multi-epitope-based peptide (MEBP) vaccine constructs have been designed against different viruses, including Middle East respiratory syndrome coronavirus (MERS-CoV) (Tahir et al., 2019), Respiratory syncytial virus (RSV) (Tahir ul et al., 2020), the Ebola virus (Ahmad et al., 2019), the Zika virus (Shahid et al., 2020), Hepatitis C Virus (HCV) (Ikram et al., 2018), HIV (Pandey et al., 2018), the BK virus (Kesherwani and Tarang, 2019), and norovirus (Azim et al., 2019), and promising results have been obtained. In comparison with single-epitope vaccines or conventional vaccines, MEBP vaccines are simple in construction, cost-efficient, time-saving, and stable, and they also have selectivity properties. MEBP vaccines concurrently induce a consequential humoral and cell-mediated immune response because of the existence of dual-purpose epitopes. Therefore, nowadays, MEBP vaccines are preferred, and various clinical trials have been carried out.

## 2. Materials and methods

### 2.1. Study design

**Figure 1:**
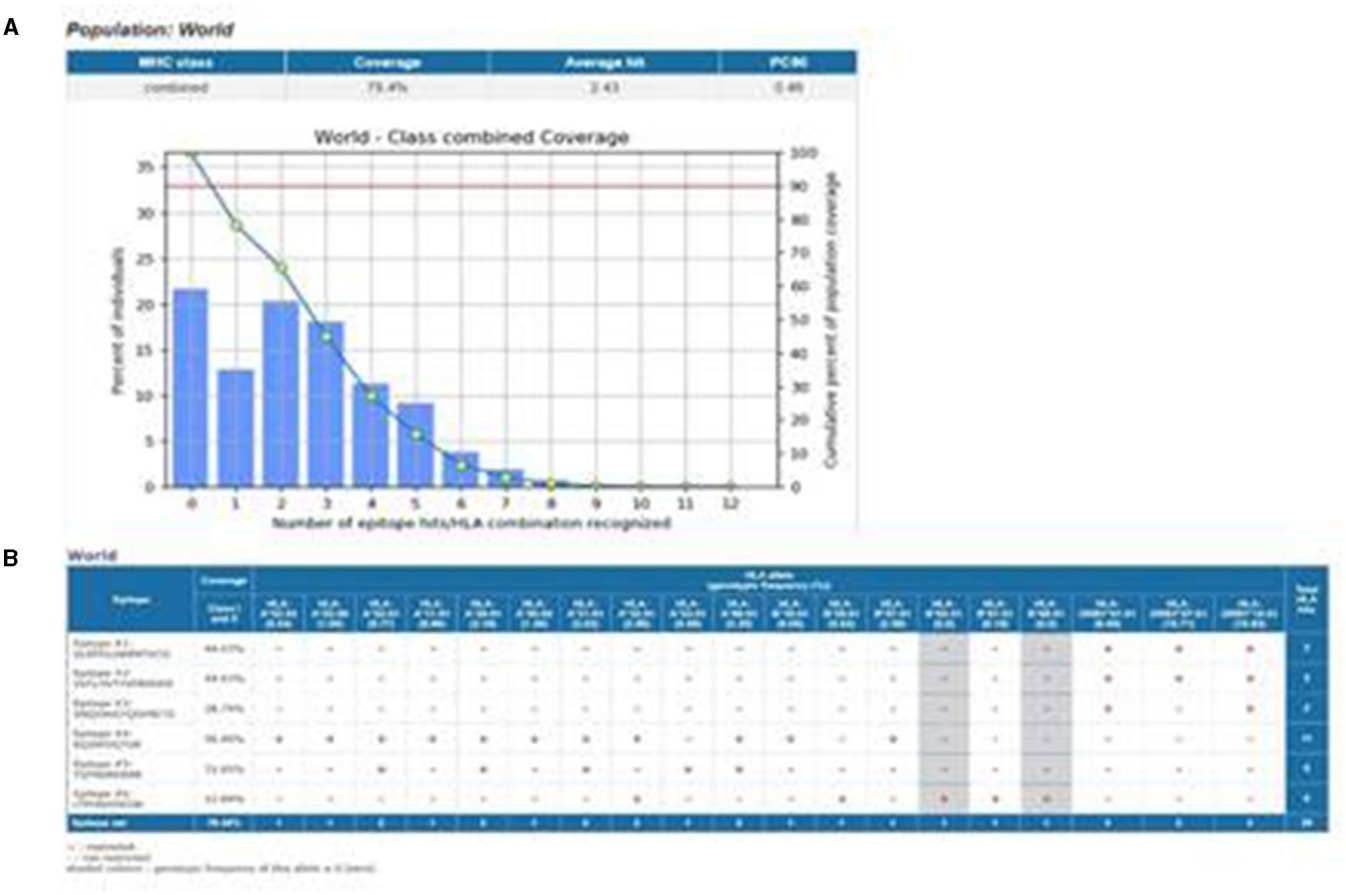
Schematic representation of the study design in this figure. **(A)** Population coverage of epitopes with the selected MHC-I and MHC-II epitopes. **(B)** Interaction of epitopes with the selected HLA alleles.

The research began by retrieving the SARS-CoV-2 spike glycoprotein (S-protein) sequence from the NCBI and introducing Delta, Lambda, Kappa, Omicron (all variants), and Iota mutations into the sequence. The sequence was further subjected to predict B-cell epitopes, CTL epitopes, and HTL epitopes. Then, antigenicity, allergenicity profiling, and population coverage analysis were performed. A multiepitope vaccine was then constructed, and its 3D model was elucidated. Finally, protein–protein docking, molecular dynamics simulation, and immune response profiling were performed. Figure 1 shows the schematic representation of the study design.

### 2.2. Spike(S)-protein sequence retrieval and the determination of antigenicity

The spike glycoprotein (S-protein) sequence was retrieved from NCBI (accession ID: NC_045512.2). Only one sequence was retrieved from NCBI to construct the peptide vaccine. Based on its antigenicity and role in viral infections, the target protein was selected for vaccine construction (Ou et al., 2020; Su et al., 2020). The Vaxijen v2.0 server was used to examine its antigenicity, which is a critical factor for stimulating natural resistance against SARS-CoV-2. Delta, Lambda, Kappa, Omicron (all variants), and Iota mutations were introduced to the sequence to study the efficiency of vaccines against different variants of SARS-CoV-2 (Singh et al., 2020).

### 2.3. Prediction of linear B-cell epitopes

In the immune system, B-cell lymphocytes play a key role as they evoke a lasting immune response against antigens. The ABCpred tool, based on an artificial neural network, was used to predict B-cell linear epitopes (Saha and Raghava, 2006).

### 2.4. Prediction of cytotoxic T-cell epitopes

Immune-Epitope-Database (IEDB) and Analysis-Resource, along with the Major Histocompatibility Complex type 1 (MHC-I) epitope prediction tool, was used to detect cytotoxic T-cell epitopes, which are important for enhancing immunogenicity. Epitopes were screened based on their frequent interactions with the HLA alleles (Peters et al., 2006).

### 2.5. Prediction of T-helper cell epitopes

The IEDB MHC-II binding prediction tool was used to predict T-helper cell epitopes. This tool was used to detect 14 mers of peptides from the S-protein. Seven HLA allele sets (DRB3 * 01:01, DRB5*01:01, DRB3 * 02:02, DRB1 * 03:01, DRB1 * 15:01, DRB1 * 07:01, and DRB4 * 01:01) were used as a reference to predict the epitopes (Wang et al., 2008).

### 2.6. IFN-γ-inducing peptide prediction

IFN-γ-inducing peptide prediction was performed using ILE-6Pred (https://webs.iiitd.edu.in/raghava/il6pred/ accessed between 3 September and 9 September 2023) for selected B-cell and T-cell epitopes. The RF-based predicted model and other common parameters were used to predict capable epitope-stimulating IFN-γ (Dhall et al., 2023).

### 2.7. Antigenicity and allergenicity profiling of epitopes

The antigenicity and allergenicity of the screened epitopes were tested using VaxiJen v2.0. (Doytchinova and VaxiJen, 2007) and AllerTOP v2.0 (Dimitrov et al., 2013), respectively. B-cell epitopes were selected based on their antigenicity and allergenicity. The screening of T-cell epitopes was conducted based on their binding affinity to HLA molecules.

### 2.8. Population coverage analysis for selected epitopes

For effective performance, the designed vaccine construct must produce a good immune response in most populations across the world. To determine the population coverage of the selected T-helper and cytotoxic T-cell epitopes, the IEDB population coverage tool was used. Nine epitopes were analyzed for population coverage, considering both Class-I and Class-II options in the calculation. The selected epitopes and their respective MHC-restricted alleles were entered and submitted (Bui et al., 2006).

### 2.9. Development of the vaccine construct

To develop MEBP vaccines using the selected epitopes, a GPGPG linker was used to join the B-cell and T-helper cell epitopes, and an AAY linker was used to join the cytotoxic T-cell epitopes. To amplify the immune reaction, β-defensin (Lioi et al., 2015) was used as an adjuvant at the N-terminal and linked with the EAAAK linker. A poly-histidine (His) tag was adjoined at the C-terminal of the vaccine construct.

### 2.10. Immune response profiling of the vaccine construct

To confirm that the vaccine does not evoke an allergic reaction, we used AllerTOP v2.0. The antigenicity parameter of a vaccine determines the extent of the body's defense against a virus. VaxiJen v2.0 was used to predict the antigenic nature of the vaccine construct.

### 2.11. Analysis of the physicochemical properties of the vaccine construct

The physicochemical properties of the vaccine construct are the salient features that are used to understand the antigenicity and stability of the vaccine. We focused our study on some physicochemical properties, such as amino acid composition, the Grand Average of Hydropathy (GRAVY) index, theoretical pI, and instability index. This was carried out using the ProtParam (Wilkins et al., 1999) online tool.

### 2.12. Prediction of the secondary structure of the vaccine construct

The secondary structure was predicted by using the online server GOR IV (Garnier, 1996). This server is based on the DPM algorithm. The DPM algorithm infers those secondary structures that use parameters, as described by Chou and Fasman.

### 2.13. 3D structure prediction

The 3D structure of the vaccine construct is a key to understanding its free energy and stability; it can be used for further studies on the vaccine's interaction with the desired target molecule. The online server RaptorX (Källberg et al., 2012) was used to predict the 3D structure of the vaccine. The modeled structure was refined by using the Swiss-PdbViewer server (Guex and Peitsch, 1997).

### 2.14. 3D structure refinement and its validation

For validation of the refined 3D structure of the vaccine construct, the ProSA web online tool (Wiederstein and Sippl, 2007) was used. ProSA calculates the gross quality score for a specific input protein structure.

### 2.15. *In-silico* verification of the vaccine construct

#### 2.15.1. Codon optimization of the vaccine

For further studies on the vaccine construct, an expression study must be performed. The codon optimization tool JCat (Grote et al., 2005) can be used to obtain the codons for a given input protein or peptide sequence. The vaccine construct's protein sequence was given as the input. This tool calculates the GC content and CAI score. The GC content more than 50% and a CAI score close to 1 are considered ideal for the maximum expression of the query sequence.

#### 2.15.2. *In-silico* expression of the vaccine

The optimized codons should then be integrated into a vector to check their *in-silico* expression efficiency. The vector opted for was *E. coli* pET-28a(+). SnapGene software was used to incorporate the sequence into the vector. This tool is used to substantiate the optimum expression of the vaccine in an expression vector.

#### 2.15.3. Molecular docking of the vaccine construct with TLR 3 and TLR 9 molecules

To analyze the interaction of the vaccine construct with TLR (Toll-like receptor) molecules, docking was carried out with various TLR molecules. From the docking result, we concluded that the binding affinity and immune response activation of the vaccine construct were most optimum with TLR 3 and TLR 9. For molecular docking, the Haddock 2.2 tool (Van Zundert et al., 2016) was used, and PyMOL Molecular Graphics System, Version 1.2r3pre, Schrödinger, LLC was used for visualization of the result. TLR extracellular domain sequences were retrieved from UniProt (the universal protein knowledge base in 2021. *Nucleic Acids Res*. 2021, *49*, D480-D489), and the 3D structure was constructed using RaptorX. The docking outcomes were visualized using PyMOL. The PDBsum server (Laskowski et al., 2005) was used for analyzing the overall pictorial structural information of the docked complexes. GROMACS (Hess et al., 2008) was used for MD simulation to check the flexibility and stability of the protein. Various graphs, such as RMSD, RMSF, H-bond, radius of gyration, and PCA, were analyzed. The RMSD graph was obtained using the program gmx rms, RMSF was obtained using gmx rmsf, radius of gyration was obtained using gmx gyrate, H-BOND was obtained from xmgrace, and PCA was obtained using gmx pca.

#### 2.15.4. Immune response profiling of the vaccine construct

Profiling of the immune response by the vaccine construct was performed using the C-immSim immune stimulator (Castiglione et al., 2021). This server works based on a PSSM algorithm to predict the immunogenic epitopes and their interactions with the immune system. For evaluating the antibodies produced, a simulation was performed with default parameters.

## 3. Results

### 3.1. Retrieval of the SARS-CoV-2 spike protein sequence

The S-protein sequence retrieved was used to predict B-cell and T-cell epitopes. VaxiJen v2.0 was used to analyze the antigenicity score of the protein, with the threshold set to 0.4, and the virus model was preferred. The protein sequence results produced an antigenicity score of 0.4646, which indicates that it is a probable antigen. The allergenicity of the protein predicted using AllerTOP v2.0 showed that the protein was non-allergen.

### 3.2. Introduction of emerging mutants into the SARS-CoV-2 reference sequence

The nssnps mutations of SARS-CoV-2 (Delta, Kappa, Lambda, and Iota variants) obtained from the co-variants online tool were introduced into the SARS-CoV-2 reference spike protein sequence to determine the extent of immune reaction against SARS-CoV-2. D614G was a commonly occurring mutation in all variants, as shown in Table 1.

**Table 1:** Mutations of SARS-CoV-2 variants.

**Sl no**.	**Kappa nssnps**	**Delta nssnps**	**Lambda nssnps**	**Iota nssnps**	**Omicron nssnps**
1	S:E154K	S:T19R	S:G75V	S:L5F	S:A67V
2	S:L452R	S:E156-	S:T76I	S:T95I	S:H69-
3	S:E484Q	S:F157-	S:R246-	S:D253G	S:V70-
4	**S:D614G**	S:R158G	S:S247-	S:E484K	S:T95I
5	S:P681R	S:L452R	S:Y248-	**S:D614G**	S:G142-
6	S:Q1071H	S:T478K	S:L249-	S:A701V	S:V143-
7		**S:D614G**	S:T250-		S:Y144-
8		S:P681R	S:P251-		S:Y145D
9		S:D950N	S:G252-		S:N211-
10			S:D253N		S:L212I
11			S:L452Q		S:G339D
12			S:F490S		S:S371L
13			**S:D614G**		S:S373P
14			S:T859N		S:S375F
15					S:K417N
16					S:N440K
17					S:G446S
18					S:S477N
19					S:T478K
20					S:E484A
21					S:Q493R
22					S:G496S
23					S:Q498R
24					S:N501Y
25					S:Y505H
26					S:T547K
27					**S:D614G**
28					S:H655Y
29					S:N679K
30					S:P681H
31					S:N764K
32					S:D796Y
33					S:N856K
34					S:Q954H
35					S:N969K
36					S:L981F

### 3.3. Prediction of B-cell epitopes and T-cell epitopes (MHC Class-I and MHC Class-II epitopes)

The selected epitopes were screened based on their antigenicity and allergenicity. Based on this selection criteria, three B-cell epitopes were picked according to the high score. Three MHC-II (Helper T-cell) and three MHC-I (cytotoxic T-cell) epitopes were selected based on the low scores. The antigenicity score of the individually selected epitopes is given in Table 2. The SNQVAVLYQGVNCTE epitope was found to have occurred frequently, containing common mutations of all variants at the D614G position (Table 2). IFN-γ is crucial to controlling viral replication. IL6-Pred results showed that the epitopes selected for vaccine construction were obtained from an epitope capable of stimulating IFN-γ.

**Table 2:** Epitopes screened for the vaccine construct.

**Sl no**.	**Types of immune cells**	**Epitopes**	**Antigenicity**
1.	B-cell	LPLVSSQCVNLRTR (Delta)	1.3003
		GVVFLHVTYPAHE (Kappa)	0.8842
		FSTFKCYGVSPTKL	0.8708
2.	T-cell (MHC-II)	VVFLHVTYVPAHEKN (Kappa)	1.0609
		SNQVAVLYQGVNCTE (Kappa, Lambda, Iota, and Delta)	0.6596
		VLSFELLHAPATVCG	0.4784
3.	T-cell (MHC-I)	RQIAPGQTGK	1.7893
		TQTNSRRRAR	0.7501
		LTPGNSSSGW (Lambda)	0.65

### 3.4. Characterization of the vaccine construct

#### 3.4.1. Formulation of the multi-epitope vaccine construct against SARS-CoV-2

At the C-terminal, β-defensin (Defensin 4A) was attached as an adjuvant. The adjuvant was linked to the B-cell epitopes with the EAAAK linker. The GPGPG linker was used to link the B-cell epitopes, the B-cell epitopes to the MHC-II binding epitopes, the MHC-II binding epitopes, and the MHC-II binding epitopes to the MHC-I binding epitopes. Three MHC-I binding episodes were linked with the AAY linker. At the N-terminal of the vaccine construct, the His tag was attached. The various linkers used in the vaccine construct are shown in Table 3.

**Table 3:** Various linkers used in the vaccine construct.

**Sl no**.	**Linkers**
1.	Adjuvant (Beta-defensin 4A)
2.	EAAAK (adjuvant to B-cell epitopes)
3.	GPGPG (B-cell epitopes, B-cell epitopes to MHC-II binding epitopes, MHC-II binding epitopes, and MHC-II binding epitopes to MHC-I binding epitopes)
4.	AAY (MHC-I binding epitopes)
5.	Histidine tag at the N-terminal

#### 3.4.2. Antigenicity and Allergenicity

The allergenicity and antigenicity analyses of the vaccine construct confirmed that the final vaccine construct was both non-allergenic and antigenic, with an antigenicity score of 0.6724 and with a threshold of 0.4 when the virus-based model was selected.

#### 3.4.3. Population coverage

A total of 79.78% of the world population exhibited an immune response to the vaccine construct, according to the IEDB population coverage online tool depicted in Figure 1A. The population coverage was calculated based on the interaction of selected epitopes with the corresponding HLA alleles (Figure 1B). This affirmed that the vaccine design was operational for most populations worldwide.

#### 3.4.4. Analysis of physicochemical properties

Numerous physicochemical properties of the vaccine construct were analyzed. Carbon, hydrogen, nitrogen, oxygen, and sulfur gave a total of 3,355 atoms, which was formulated as C1081H1668N306O288S12. The isoelectric point of the vaccine construct was 9.59, suggesting that it is basic. The extinction coefficient was measured at 280 nm in water. The GRAVY index (-0.042) and the instability index (36.80) indicated the vaccine construct's stability. The aliphatic index was found to be 73.07. The half-life was estimated to be 30 h for mammalian red blood cells (*in vitro*), which indicates the total time it took to disappear after it was synthesized in the cell.

#### 3.4.5. Secondary structure prediction

**Figure 2:**
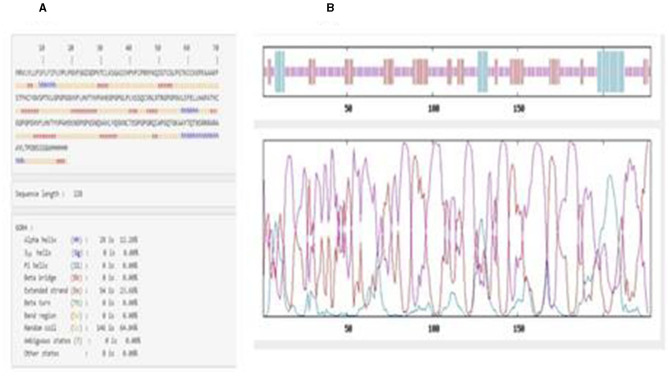
**(A)** Amino acid sequence showing the positions of helices, turns, and coils in the vaccine construct. **(B)** Graphical overview of secondary structure characteristics.

The secondary structure results from GOR IV showed that the vaccine construct was composed of an alpha-helix (12.28%), random coil (64.04%), and extended strand (23.68%), as shown in Figure 2A. The graphical overview shows the probability of formation of different secondary protein structures along the sequence in Figure 2B.

#### 3.4.6. 3D structure prediction and structure refinement

**Figure 3:**
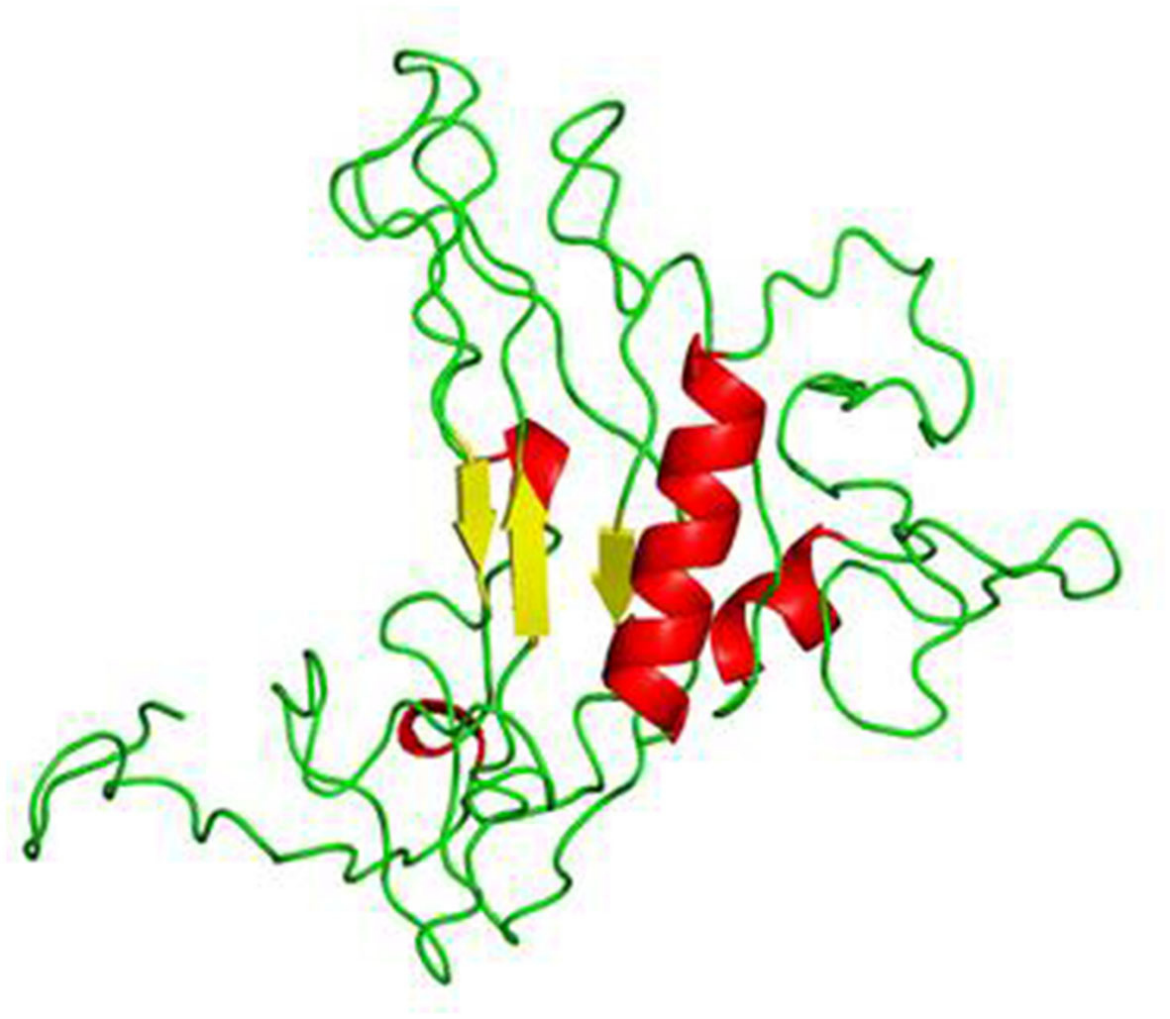
3D structure of the vaccine construct formed by RaptorX.

The 3D structure of the vaccine construct was built using RaptorX. The predicted results showed the contact-based distance matrices, which were ≤ 8Å. The properties showed the percentage of helices, coils, beta-turns, bridges, bends, disordered and ordered regions, and the solvent accessibility of the protein. The structure was refined using the Swiss-PdbViewer to optimize the 3D structure. The refined 3D structure of the vaccine construct visualized in PyMOL is shown in Figure 3.

#### 3.4.7. 3D structure validation

**Figure 4:**
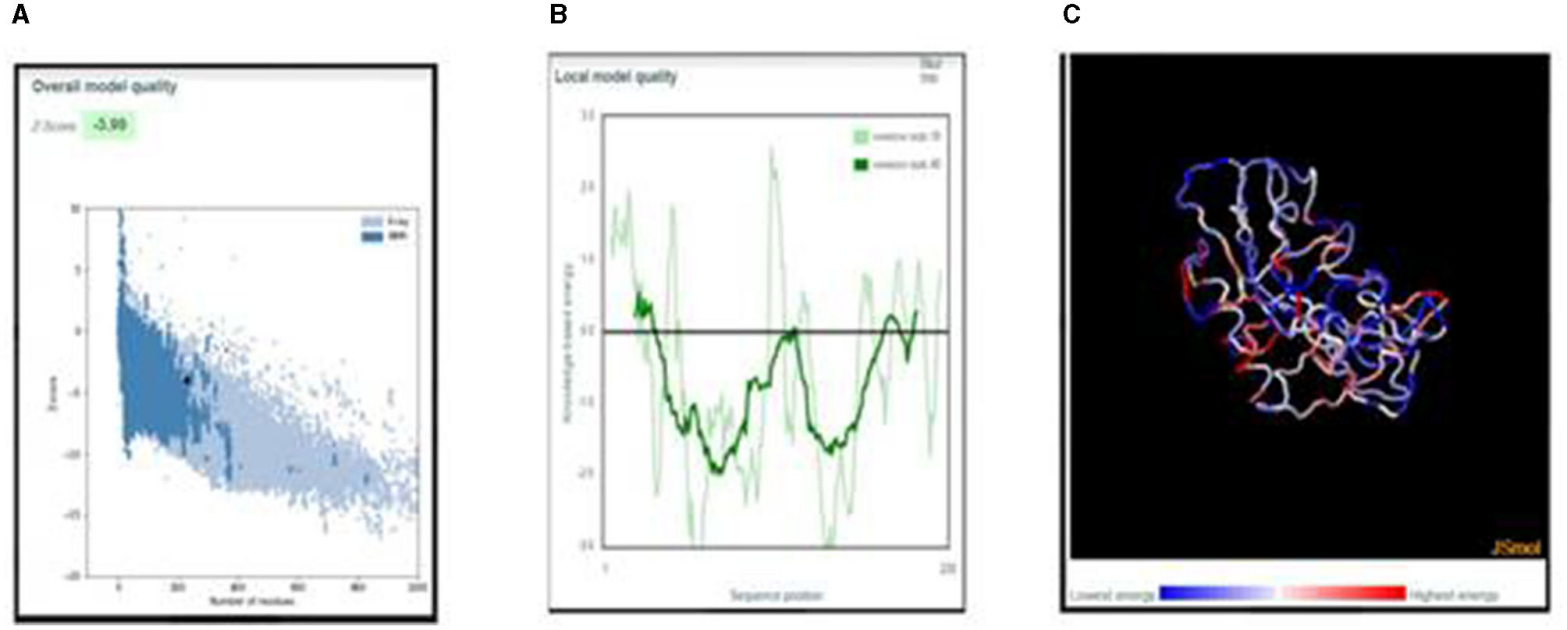
**(A)** Z-score plot of the refined structure representing the quality of the vaccine construct in the NMR region. **(B)** Local model quality representing the energies as a function of amino acid sequence position. **(C**) 3D structure of the vaccine construct with energy representation.

ProSa was used for validating the 3D structure. The overall model quality was in the NMR region. In the local model quality assessment, a window size of 40 residue fragments was chosen; most residues had negative values, indicating a good-quality structure. The Z-score of the refined structure was −3.99, which was close to the range of scores for native proteins of similar size, as shown in Figure 4. The free energy graphical representation indicates a range of amino acids in the structure (Figure 4B), with the highest energy marked in red and the lowest energy marked in blue, as shown in Figure 4C.

#### 3.4.8. Codon optimization and *in-silico* cloning

**Figure 5:**
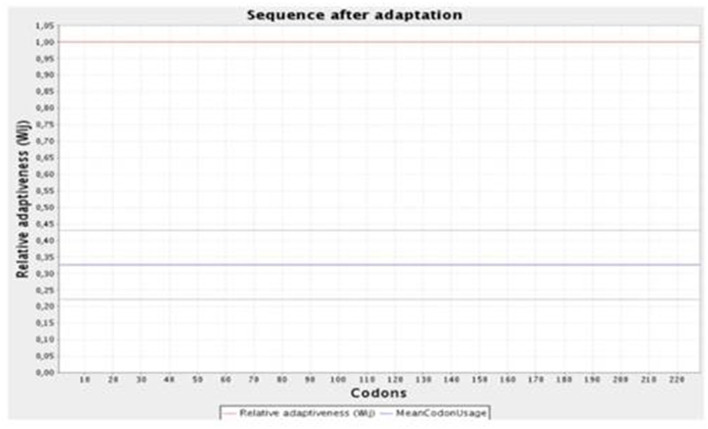
Graphical representation of the codon optimization of the vaccine construct in the *E. coli* K12 host organism.

**Figure 6:**
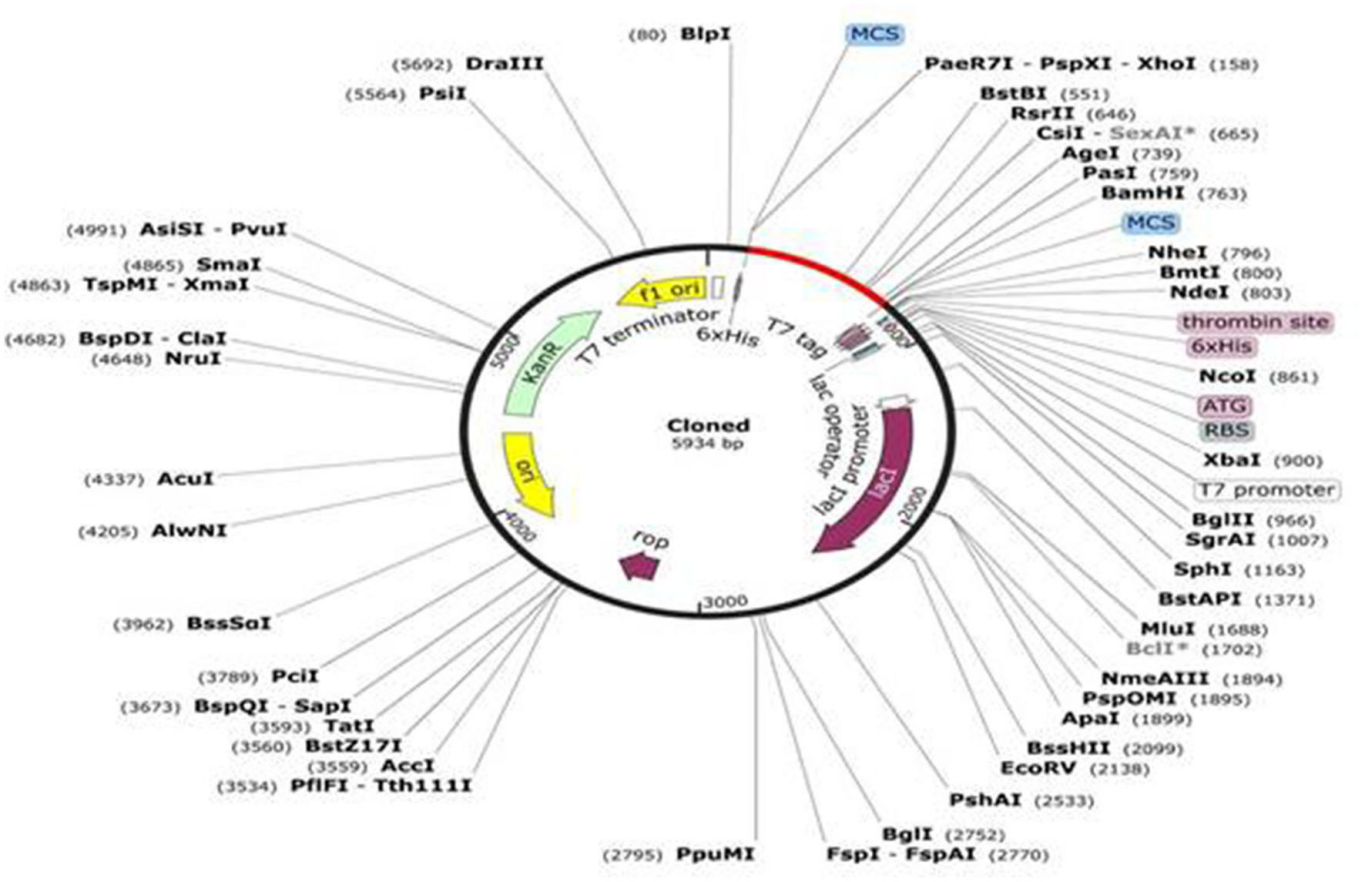
*In-silico* cloning of the vaccine construct in the pET-28a (+) expression vector in which the red part indicates the insert and the remaining is the vector genome.

The JCat online tool was used to optimize the codons of ~694 nucleotides of the vaccine construct. The codon optimization index (CI) was 1.0, and its GC content was 57.45%, which affirmed a positive response in the host *E. coli* K12 organism. At the C and N terminals of the adapted codon sequence, restriction sequences of XhoI and BamHI restriction enzymes were added (Figure 5). The adapted codon sequence cloned in the pET-28a(+) expression vector using SnapGene software indicated that the cloned vector of the vaccine construct can be further used for the transformation of host cells (Figure 6).

#### 3.4.9. Molecular docking of the vaccine construct with TLR receptors

**Figure 7:**
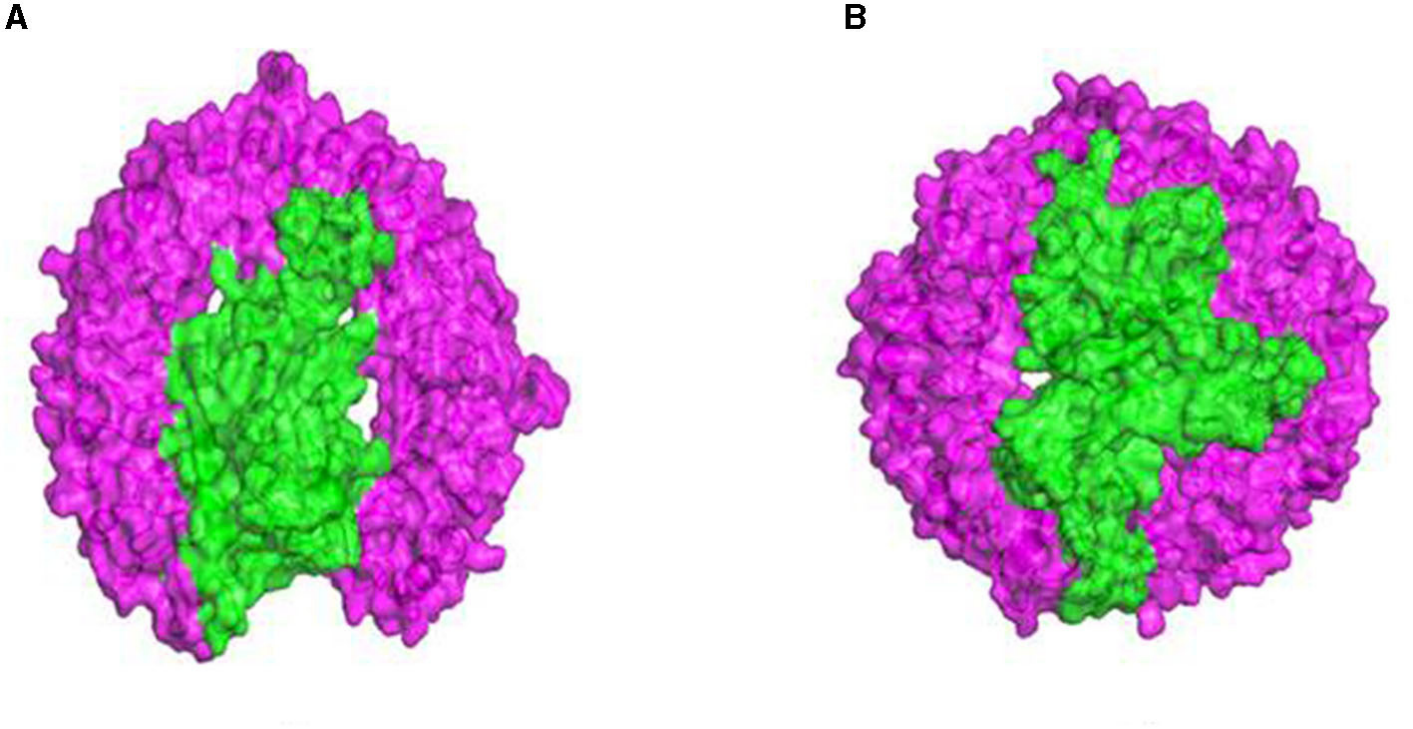
**(A)** Docked complex of the vaccine construct and TLR 3 molecule. **(B)** Docked complex of the vaccine construct and TLR 9 molecule.

The 3D structures of TLR molecules (TLR 3 and TLR 9) were predicted using RaptorX. The secondary structure properties predicted by PDBsum using RaptorX models of the TLR molecules showed good stability and conformation, with the potential for further studies to be carried out for docking purposes. These models were again selected based on their low score and high rank. An analysis of the docking of the vaccine construct with TLR 3 (Figure 7A) and TLR 9 molecules was carried out using the Haddock 2.2 server (Figure 7B). A secondary structure analysis of the TLR-vaccine construct docked complex was performed using PDBsum.

**Figure 8:**
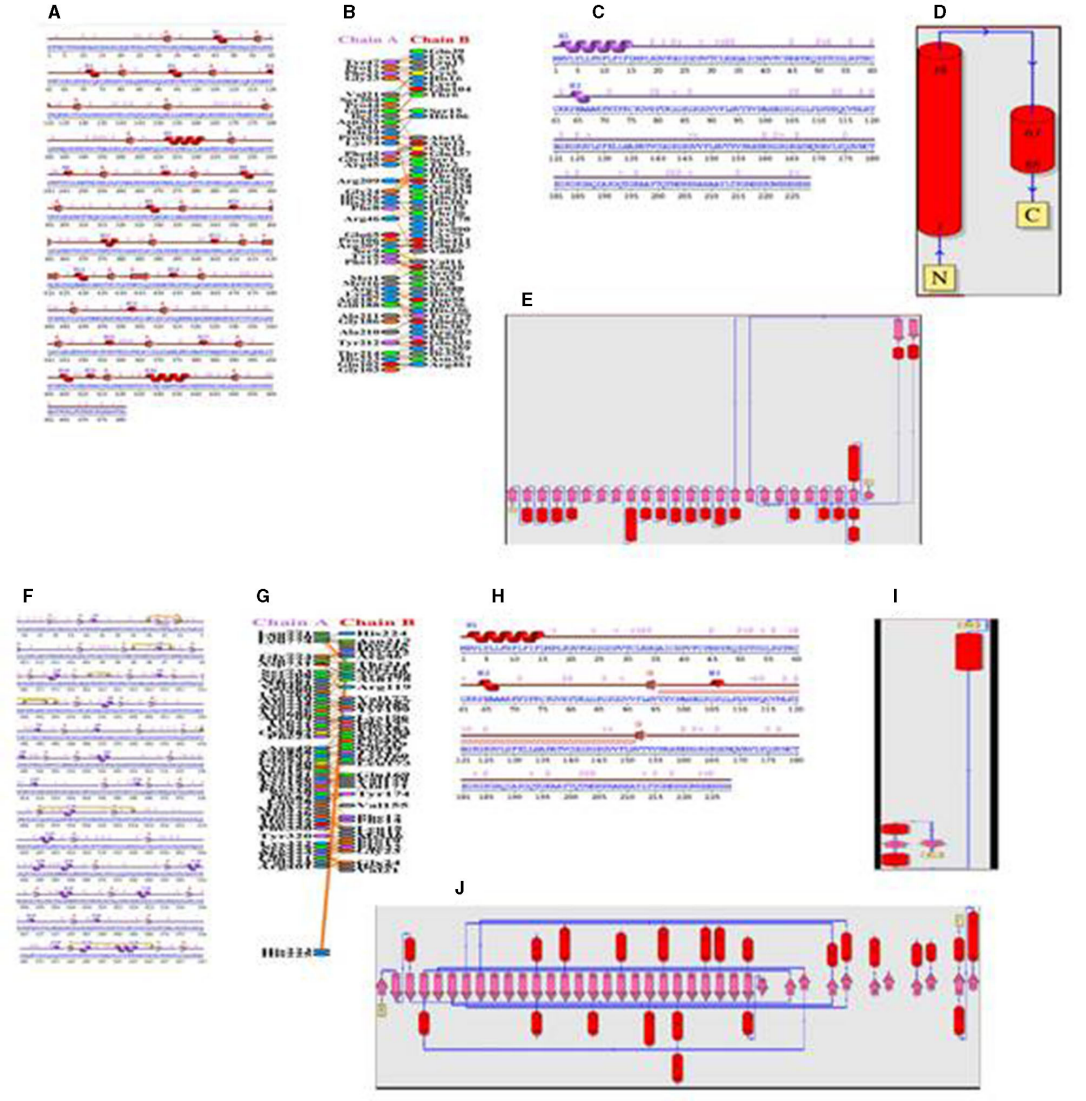
**(A)** Secondary structure of the TLR 3-vaccine construct complex. **(B)** Interaction of the vaccine with TLR 3. **(C)** Secondary structure of the vaccine construct. **(D)** Topology of the vaccine construct. **(E)** Topology of the TLR 3 molecule. **(F)** Secondary structure of the TLR9-vaccine construct complex. **(G)** Interaction of the vaccine with TLR 9. **(H)** Secondary structure of the vaccine construct. **(I)** Topology of the vaccine construct. **(J)** Topology of the TLR 9 molecule.

TLR 3 (chain B) comprised 681 a.a, containing 2 sheets, 7 beta-alpha-beta units, 1 beta bulge, 27 strands, 20 helices, 4 helix–helix interactions, 115 beta-turns, and 35 gamma-turns (Figures 8A, B). The vaccine construct (chain A) comprised 228 a.a, containing 2 helices, 33 beta-turns, and 20 gamma-turns, showing the wiring diagram of TLR 3 and the vaccine construct and their topology (Figures 8C–E). The TLR 9 (chain A) comprised 779 a.a, containing 6 sheets, 4 beta-alpha-beta units, 1 beta-hairpin, 1 beta-bulge, 38 strands, 23 helices, 4 helix–helix interactions, 133 beta-turns, 20 gamma-turns, and 7 disulfides (Figures 8F–H). The vaccine construct (chain B) comprised 228 a.a, containing 1 sheet, 1 beta-hairpin, 2 strands, 3 helices, 30 beta-turns, and 23 gamma-turns, which shows the wiring diagram of TLR9 and the vaccine construct and their topology (Figures 8I, J).

The protein–protein interface represents different types of interactions between chain A and chain B, and each interaction is depicted according to the key in Figures 8B, G. The area of each circle is proportional to the surface area of protein chain A and protein chain B. The degree of the interface region on each chain is depicted by a black wedge, whose size indicates the surface area of the interface region. The statistics of this interface region are given in Tables 4, 5. The interactions with residues across the interface show that the number of H-bond lines between any two residues represents the number of possible hydrogen bonds between them, and for non-bonded contacts, the width of the striped line is equal to the number of atomic contacts.

**Table 4:** Bonded and non-bonded contact results from PDBsum.

**Secondary structure parameters**	**TLR 3**		**TLR 9**	
	**Chain A**	**Chain B**	**Chain A**	**Chain B**
No. of interface residues	43	53	54	49
Interface area (Å2)	2,556	2,335	2,601	2,654
No. of salt bridges	4		1	
No. of disulfide bonds	-		-	
No. of hydrogen bonds	24		18	
No. of non-bonded contacts	265		303	

**Table 5:** Haddock scores of TLR 3 and TLR 9.

**Clusters**	**TLR 3**		**TLR 9**	
	**Haddock score**	**Z-score**	**Haddock score**	**Z-score**
Cluster 7	–**102.1** **+/**–**13.3**	–**2**	**184.5** **+/**–**41.1**	**0.5**
Cluster 4	−28.8 +/- 30.8	−0.1	198.3 +/- 7.7	0.8
Cluster 5	4.1 +/−25.5	0.8	162.3 +/−12.4	0
Cluster 2	−72.0 +/−19.3	−1.2	106.9 +/−28.8	−1.4
Cluster 1	−6.5 +/−7.4	0.5	134.0 +/−20.5	−0.7
Cluster 6	−63.3 +/−22.2	−1	178.7 +/−20.1	0.4
Cluster 11	-	-	225.8 +/−60.2	1.5
Cluster 8	-	-	147.6 +/−19.0	−0.4
Cluster 3	7.4 +/−12.6	0.9	207.4 +/−13.4	1.0
Cluster 9	-	-	90.2 +/−8.7	−1.8
Cluster 14	−16.1 +/−42.5	0.3	-	-
Cluster 12	0.4 +/−52.4	0.7	-	-
Cluster 13	10.0 +/−4.6	1.0	-	-

Bold values indicate best cluster score.

#### 3.4.10. MD simulation

MD simulation confirmed the thermal stability of the vaccine construct from RMSD (root mean square deviation), RMSF (root mean square fluctuation), RG (radius of gyration), H-bond, and PCA (principal component analysis). In all the plots, the TLR 3-vaccine complex is depicted in black, and the TLR9-vaccine complex is depicted in red.

### 3.5. RMSD

**Figure 9:**
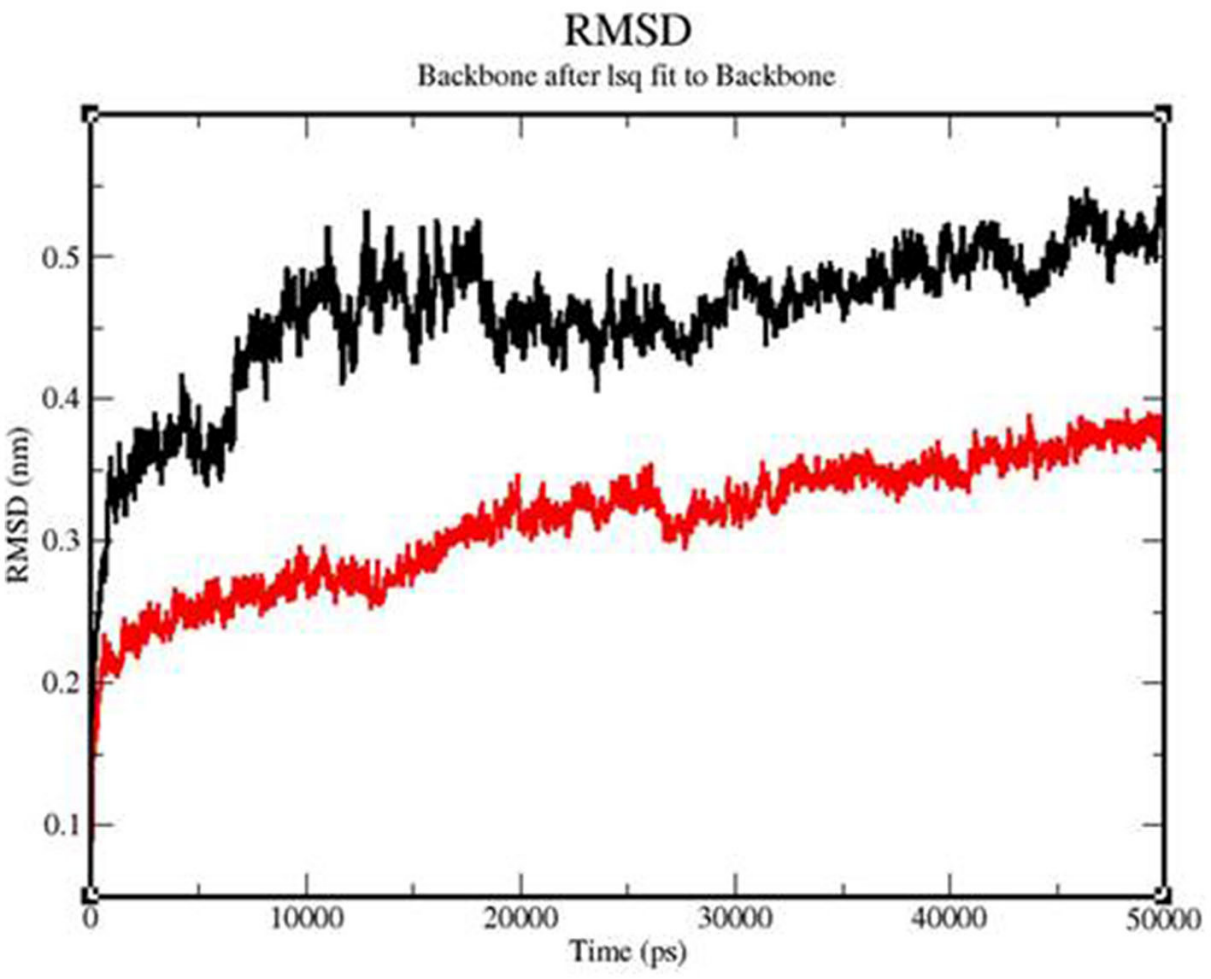
Backbone RMSD of the TLR3-vaccine construct complex (black) and TLR9-vaccine construct complex (red) as a function of time.

The backbone RMSD value varied from 0.3 to 0.54 for the TLR 3-vaccine complex and from 0.23 to 0.39 for the TLR 9-vaccine complex (Figure 9). An extensive study showed that after 6515.06 ps, the TLR 3-vaccine complex showed a greater RMSD value, up to 0.51 nm. At 11725.7 ps, it decreased to 0.41 nm. It showed an increase to 0.53 nm yet again at 12837.3 ps, and it showed a few fluctuations in the RMSD value up to 50,000 ps. The TLR 9-vaccine complex showed an RMSD value converging to 0.25 nm at 12837.3 ps. The RMSD value showed a slight increase to 0.35 nm at 26107.1 ps and a slight decrease to 0.29 nm at 27705.1 ps. It continued to converge in the range of 0.33 to 0.39 nm until 50,000 ps. The greater the RMSD value, the lesser the stability of the protein and vice versa.

### 3.6. RMSF

**Figure 10:**
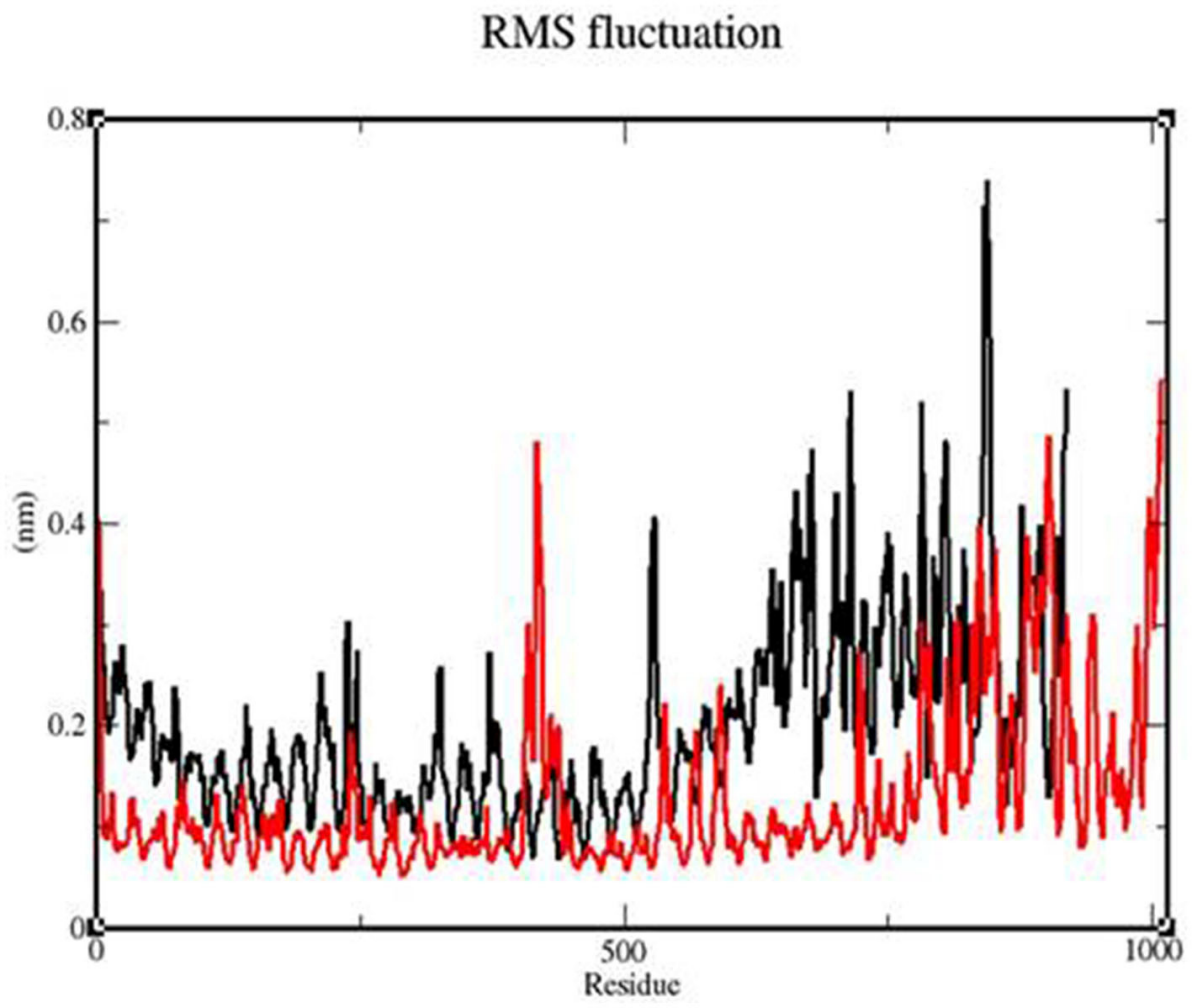
RMSF profile of the TLR3-vaccine construct complex (black) and the TLR9-vaccine construct complex (red).

To determine whether the TLR-vaccine complex affects the dynamic behavior of residues, the RMSF values of the TLR 3-vaccine complex and TLR 9-vaccine complex were compiled (Figure 10). The TLR 9-vaccine complex showed fluctuations in the range of 420–1,010 residues from 0.4 to 0.53 nm. The TLR 3-vaccine complex showed higher fluctuations in the range of 525–846 residues from 0.4 to 0.7 nm. Based on the above-mentioned fluctuations, the TLR 9-vaccine complex showed less flexibility than the TLR 3-vaccine complex.

### 3.7. Radius of gyration

**Figure 11:**
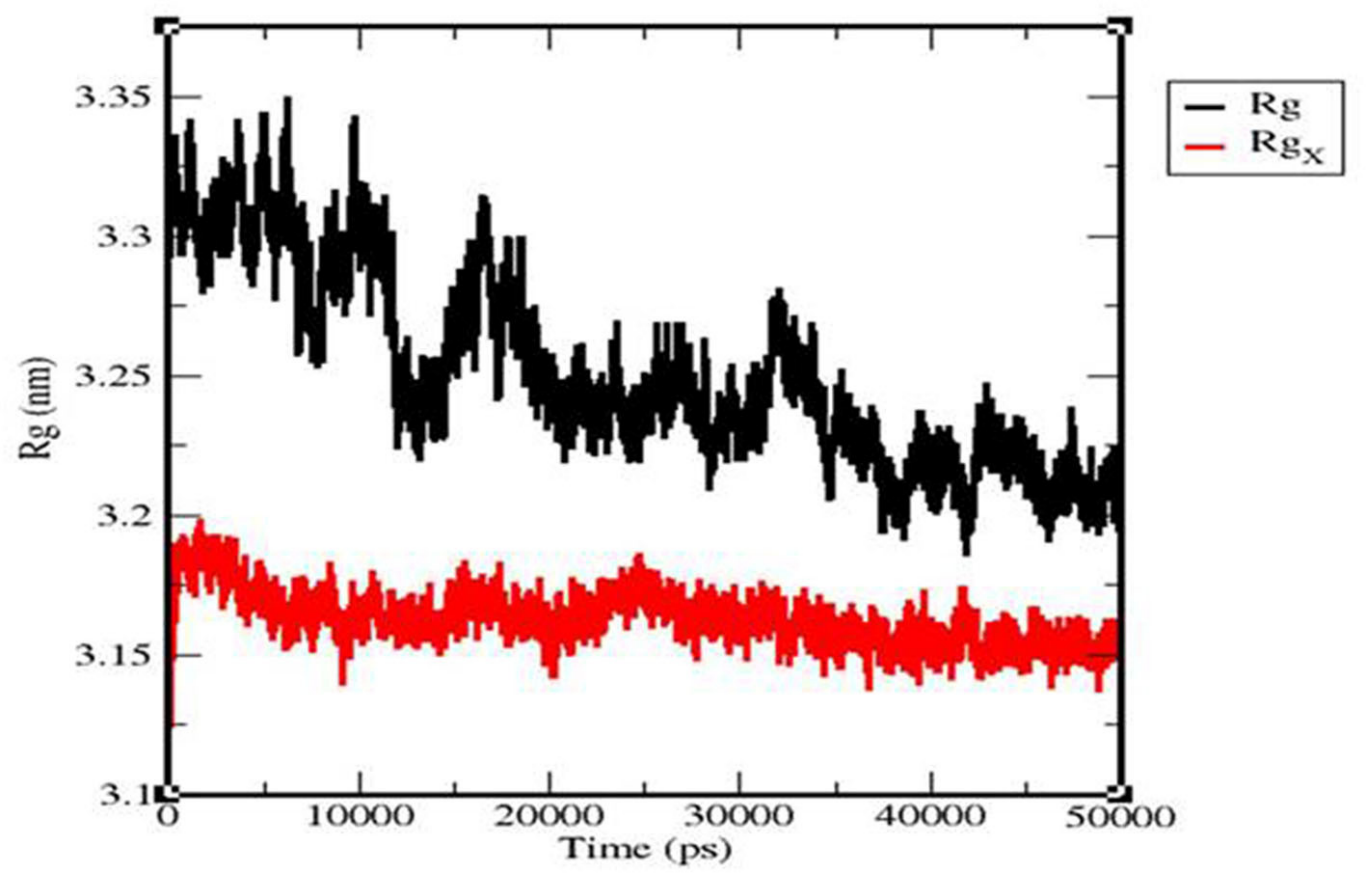
Radius of the gyration plot of the TLR3-vaccine construct complex (black) and TLR9-vaccine construct complex (red).

The Rg value of the TLR 3-vaccine complex was primarily observed to fluctuate in a descending manner from 3.35 to 3.22 nm. The TLR 9-vaccine complex maintained a constant Rg value, with a few fluctuations between 0 and 25,000 ps at 3.18 nm, and converged up to 50,000 ps from 3.15 to 3.16 nm (Figure 11).

### 3.8. H-Bonds

**Figure 12:**
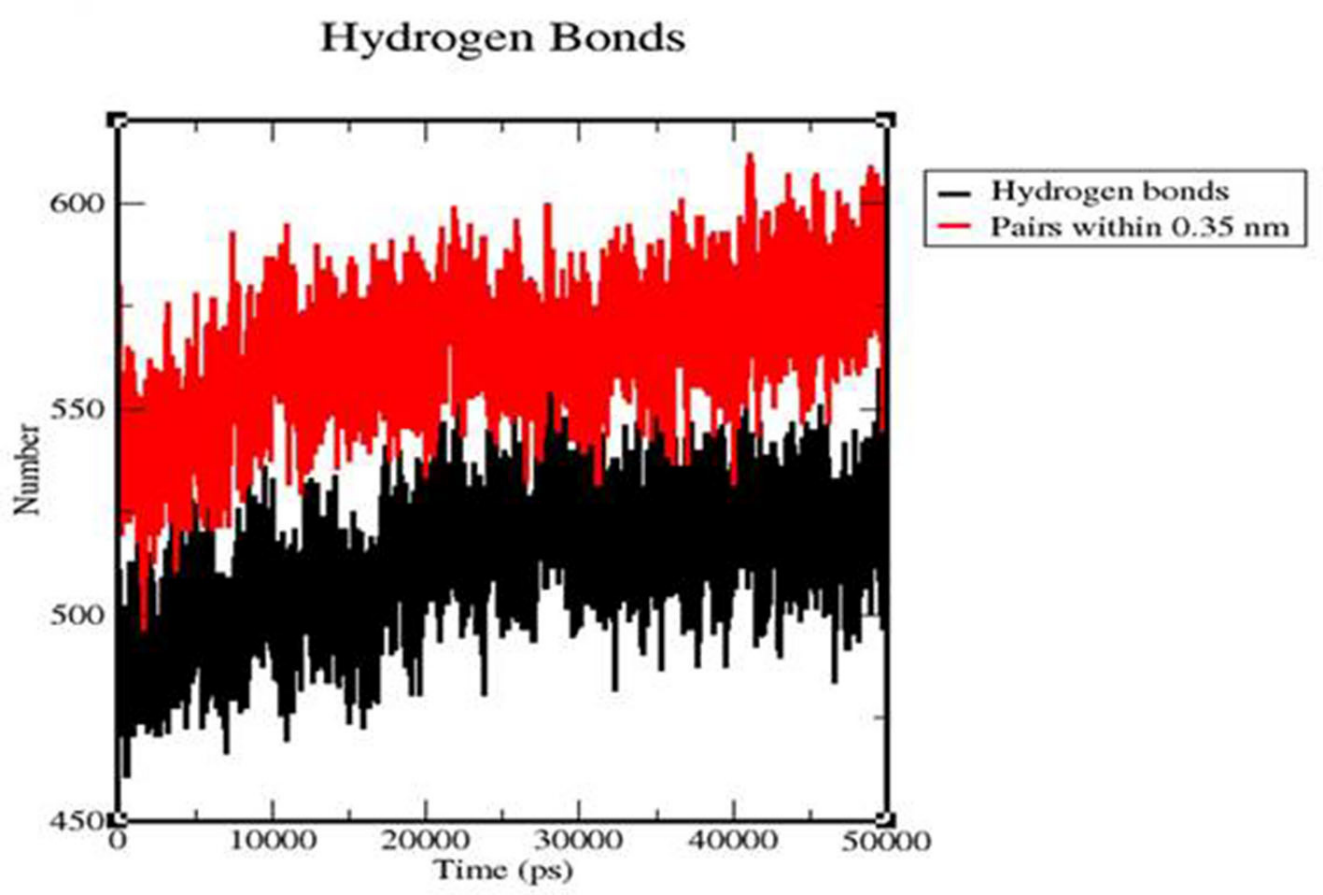
Time evolution of intra-molecular hydrogen bonds involved in the TLR3-vaccine construct complex (black) and TLR9-vaccine construct complex (red).

We calculated the intermolecular H-bonding for the TLR 3-vaccine complex and TLR 9-vaccine complex throughout the simulation time. The intermolecular hydrogen bonding in the protein plays a crucial role in stabilizing the protein. The higher the number of H-bonds, the more the stability of the complex. The number of H-bonds in TLR 3 was in the range of 475–544 within 80–28,012 ps, and the number remained stable between 27,089 and 50,057 ps with the range of 525–522. The number of H-bonds in TLR 9 increased in the range of 496–595 within 1,581–10,930 ps, and the number of H-bonds was stable within the range of 561–564 between 10,237 and 31,705 ps (Figure 12).

### 3.9. PCA

**Figure 13:**
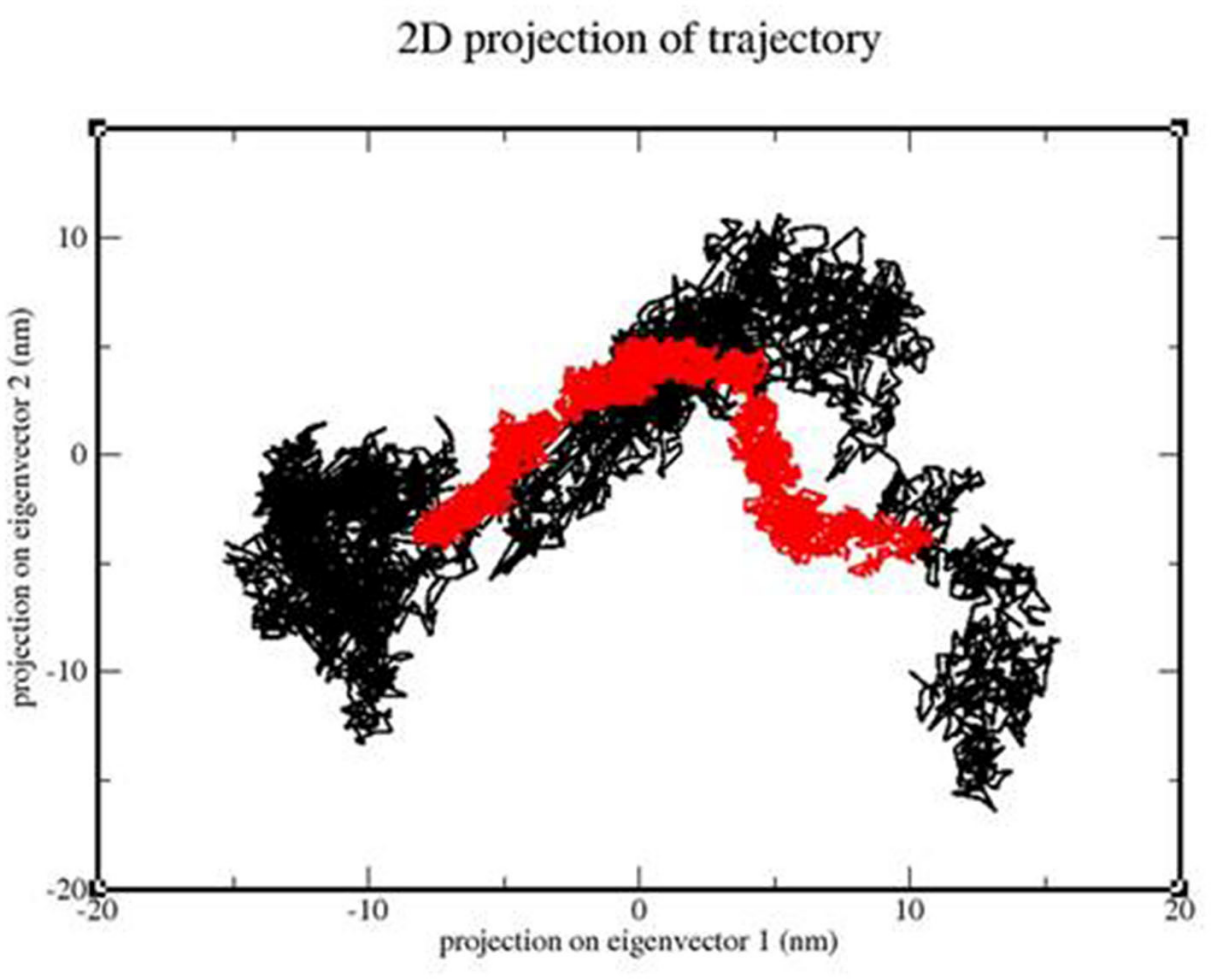
2D projection of the trajectory of the TLR3-vaccine construct complex (black) and TLR9-vaccine construct complex (red).

We calculated the PCA values for the TLR 3-vaccine complex and the TLR 9-vaccine complex throughout the simulation time. This calculation helped determine the stability of the complex. The lower the distribution, the higher the stability. TLR 3 showed more conformational changes due to its extensive distribution, whereas TLR 9 showed less conformational changes due to its structure (Figure 13).

#### 3.9.1. Immune simulation

**Figure 14:**
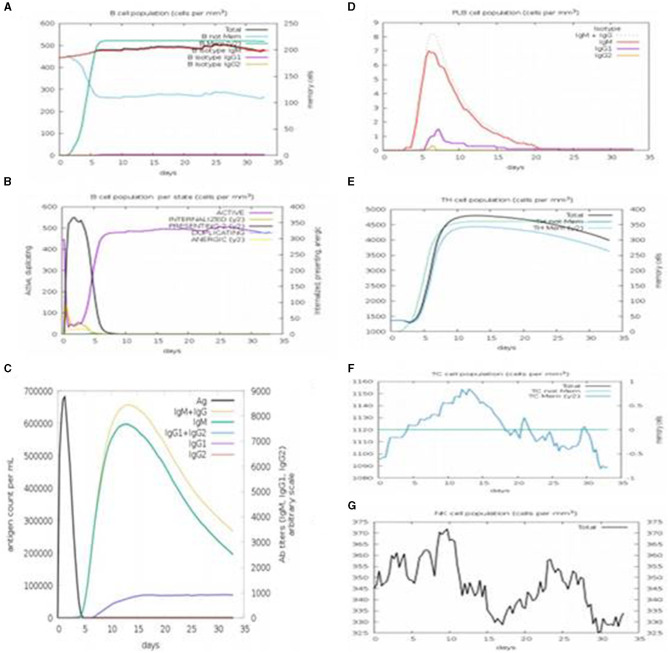
C-immSim results. **(A)** The immune response profiling of the vaccine constructed with a single dose of injection. **(B)** It shows the antibody titer according to the antigen count per milliliter. **(C)** PLB cell count with the vaccine construct. **(D)** Population of T-helper cells and memory cells produced. **(E)** Population of cytotoxic T-cells and memory cells produced. **(F)** Count of natural killer (NK) cells with the vaccine construct. **(G)** NK Cell population.

Immune simulation was performed using C-immSim. A single dose of antigen for HLA alleles was administered. Various graphs were generated to show the production of different immune cells in the body and the antigen count per milliliter that triggered antibody production in the body. The total B-cell count, memory cells, and the production of isotypes IgM, IgG1, and IgG2 were measured. The B-cell population per entity state, which showed the counts of active, presenting, and internalized antigens, as well as duplicating and anergic states of B-cells, was evaluated. The plasma B-cell (PLB cell) count per isotype (IgM, IgG1, and IgG2) was calculated. The total count of CD4 T-helper cells, T-regulatory cells, CD8 T-cytotoxic cells, and memory cells, along with the count per entity state indicating active, resting, anergic, and duplicating state, was calculated. The total count of natural killer cells was calculated. Dendritic cells (DC), macrophages (MA), and epithelial cells (EP), presenting the total count of antigenic peptides per entity state, were plotted. The concentration of cytokines and interleukins was assessed using plots recorded for 35 days (Figures 14A–G).

## 4. Discussion

A life-threatening viral disease called COVID-19 caused a global pandemic in early 2020 due to its highly evolving nature and transmission rate. Numerous drugs and treatments were available, but none of them were established as effective in suppressing it. Vaccines are a single effective tool that can be used to fight against this deadly infection. The typical approach to vaccine development involves problems associated with its efficiency and expression. To address these problems, the latest approach of an “epitope-based vaccine” is promising compared to using the entire protein used as a vaccine. The accessibility of advanced computational tools and proteome information concerning an organism simplifies the creation of a subunit-based vaccine, which is antigenic and capable of eliciting an immune response. Immunoinformatics plays a significant role in the development of a stable, effective, and safe vaccine for human administration.

The genome of SARS-CoV-2 is 29,903 bp long with a linear ss-RNA. This study targeted the surface glycoprotein, the spike protein of SARS-CoV-2 (CDS: 21563.25384), which is significant for viral infections in humans due to its high antigenicity. Various computational tools were used for selecting the antigenic region of the protein to develop a vaccine. Before selecting the epitopes, mutations of SARS-CoV-2 (Delta, Kappa, Lambda, and Iota) obtained from the co-variants online tool were introduced into the spike reference protein sequence to understand the extent of immune response that can be caused in the host body. The SNQVAVLYQGVNCTE epitope contains the mutation D614G, which is a common mutation in all variants that can produce a good immune response. T-cell and B-cell epitopes of the S-protein containing the mutations of the variants were used for vaccine construction. The B-cell and T-cell epitopes were selected using the ABCpred tool and the IEDB online server, respectively, and their antigenicity and allergenicity were tested using VaxiJen v2.0 and AllerTOP v2.0, respectively.

Variations between Omicron and Delta variants have been utilized to design multi-epitope-based vaccines (Dawood, 2022). In this study, variations among four variants, Delta, Kappa, Lambda, and Iota, were utilized to design a multi-epitope-based vaccine. The interaction of the vaccine construct designed in this study with TLR showed better results with TLR 9. Contrary to this finding, a vaccine construct based on the Omicron and Delta variants showed better results with TLR 3 (Dawood, 2022).

The IEDB population coverage tool results showed that 79.78% of the world population was covered by the vaccine construct, based on its interactions with multiple HLA alleles. Adjuvant, B-cell, and T-cell epitopes, as well as a Histidine tag, were built into the vaccine and held together by linkers (EAAAK, GPGPG, and AAY). There are three B-cell epitopes, three MHC Class-I binding epitopes, and three MHC Class-II binding epitopes. The T-cell epitopes were selected based on their binding affinity for MHC-I and MHC-II molecules. Adjuvant β-defensin was attached at the C-terminal to protect the vaccine from degradation, to enable efficient binding to the TLR molecules, and to ensure that the vaccine acts as an agonist and enhances the body's natural defense. The adjuvant β-defensin was linked with B-cell epitopes using the EAAAK linker. The GPGPG linker was used to link B-cell epitopes and cytotoxic T-cell epitopes with MHC Class-I binding epitopes using the AAY linker. To facilitate the cloning of the vaccine construct into vectors, the His tag was attached at the N-terminal of the vaccine construct. The final vaccine construct was both non-allergenic and antigenic and proved to be an effective candidate for providing immunity. The vaccine construct was 228 amino acids long, with a molecular weight of 23943.73 Da. The isoelectric point for the vaccine construct suggested that it is basic. Extinction coefficient refers to the absorption of light in a medium.

The average extinction coefficient was measured to be 17732.5. The extinction coefficient can be used for further spectral UV analysis studies on the vaccine construct. The GRAVY index and the instability index specified the stability of the vaccine construct. The GRAVY index of the vaccine construct was −0.042. Proteins with a GRAVY index <0 are more likely to be hydrophilic (Atapour et al., 2020). An instability index of <40 is considered a stable protein. The instability index of the vaccine construct was 36.80, indicating its stability. The relative volume occupied by aliphatic side chains (alanine, valine, isoleucine, and leucine) is referred to as the aliphatic index. It enables further studies on the thermostability of globular proteins. The aliphatic index in the range of 66.5–84.33 indicates the thermal stability of the protein (Panda and Chandra, 2012).

The aliphatic index of the vaccine construct was 73.07, indicating a thermally stable construct. The short half-life of the peptide suggested that it can remain viable for an adequate span of time to generate a potent immune response. The RaptorX server was used to assess the 3D structure of the vaccine construct, and it was refined with the Swiss-PdbViewer Web server for energy minimization. The ProSA online server was used to evaluate the overall and local quality of the refined model and to predict the free energy graphically. The overall model quality indicates the quality of models from distinct sources, such as NMR and X-ray. The local model quality gives energies as a function of amino acid sequence positions. Single-residue energies contain extensive fluctuations and have confined values for model evaluation; therefore, a window size of 40 residue fragments was chosen. Free energy representation indicates the increasing order of residue energy from blue to red.

After refinement and validation of the vaccine model, the quality of the predicted model was observed to be standard at 85%, as more residues were found in favored regions. Codon adaptation showed an increase in the expression of the MEBP vaccine insert in strain K12 of *E. coli*. The protein–peptide interaction showed the significant binding affinity of the MEBP vaccine construct with TLR 3 and TLR 9. This indicates that the immune system will be able to recognize the molecular patterns of the virus and trigger an immune reaction. Secondary structure scrutinization of docked complexes using PDBsum helped obtain the number of turns, helices, and strands present in individual TLRs and vaccine construct chains, indicating the flexibility of the vaccine complex. It also predicted the protein–protein interface and the bonds formed in the interface region. This included information about the area occupied by the interface and the residue interactions with various bonds (H-bonds, disulfide bonds, non-bonded contacts, and salt bridges) between the TLR chain and the vaccine chain, indicating the stability and extent of interaction within the docked complex.

Molecular dynamics simulation: MD simulation was performed for the TLR 3-vaccine complex and the TLR 9-vaccine complex to comprehend the structural consequence of the MEBP vaccine construct under the simulation condition. Five parameters, RMSD, radius of gyration, H-bonds, RMSF, and PCA, were evaluated. RMSD is normally used to indicate the structure approaching an equilibrium state. It is a measure of distance; the higher the RMSD value, the lower the stability of the complex and vice versa. The trajectory in the graph indicated structural stability at ~50 ns. The RMSF indicated the variance of atoms about its normal position. This helped us understand the flexibility of different regions of the complex. According to the results obtained, the TLR9-vaccine complex showed less flexibility than the TLR3-vaccine complex. This indicates that the TLR9-vaccine complex is more stable than the TLR3-vaccine complex. The radius of gyration can be used to understand the shape of the molecule at any instant in time. A stable Rg value of the TLR9-vaccine complex indicated a stable fold. H-bonding is a property that calculates the total number of hydrogen bonds, including those within the peptide or between the peptide and the surrounding solvent. The presence or absence of an H-bond is traced from the distance between a donor-H-acceptor angle and the donor-H-acceptor pair. The TLR 9-vaccine complex showed a higher number of hydrogen bonds, indicating higher stability. PCA gives the fluctuations in the total number of amino acid residues, where the lower the fluctuation, the more stable and compact the protein structure. Hence, the TLR 9-vaccine complex is more stable.

Immune simulation of the MEBP vaccine construct showed that it can trigger an outstanding immune reaction by producing pathogens and neutralizing antibodies and other immune cells after administration. For a maximum antigen count per milliliter (700,000/ml), the antibody concentration in terms of IgM (2,300/ml), IgG1 (1,000/ml), IgG2 (0/ml), IgG1+IgG2 (up to 3,000/ml), and IgM+IgG (3,200/ml) indicated a good response. It can also be concluded that both humoral and cell-mediated immune responses were triggered. Computational and immunoinformatics tool studies verified that the vaccine construct is stable, highly effective, and safe as a target for an advanced vaccine against SARS-CoV-2. Furthermore, the accuracy and effectiveness of vaccines can be understood through *in-vitro* studies.

## 5. Conclusion

In our study, we utilized reverse vaccinology and immunoinformatics approaches to design a multi-epitope peptide-based vaccine construct for immunization against the emerging variants of SARS-CoV-2, such as Alpha, Beta, Delta, Gamma, all Omicron variants, Kappa, Lambda, Iota, and Epsilon. This multi-epitope-based vaccine construct was found to have the potential to generate both B-cell and T-cell-mediated immune responses against COVID-19, thereby creating a strong resistance inside the body. Various online tools, servers, and *in-silico* approaches were used to predict epitopes, antigenicity, allergenicity, and population coverage, to analyze the secondary structure, generate a 3D model, and ensure its validation and simulation in the human body. The molecular interaction between TLR molecules and the vaccine construct was studied.

## Data availability statement

The original contributions presented in the study are included in the article/supplementary material, further inquiries can be directed to the corresponding author.

## Author contributions

KK designed the study and wrote the protocol and the first draft of the manuscript. KK, YK, SB, SS, TM, KS, BA, MS, DR, SMS, and MM conceived the study and contributed to the writing and editing of the manuscript. All authors read and approved the final manuscript.
